# With regard to the reform of the law of medical ethics

**Published:** 2015-12-30

**Authors:** Fernando Sanchez Torres

**Affiliations:** President of the Colombian Institute of Bioethics Studies. Ex-magistrate of the Tribunal Nacional de Etica Medica, Cali, Colombia

The law or code of medical ethics must be understood as the moral navigation chart of medicine practitioners, as what it is aimed with it is to help them not to get lost during the course of their work, pointing them out the right way to act with their patients and the community. That guide is the "objective moral", that is, the set of rules that dictates the society for its components, including the doctors themselves.

As such, the code of medical ethics has the patient as its first beneficiary, which is the rationale of medicine. What is aimed is that the doctor be a pledge of warranty for the care of the health and life of people, interpreting that the former and the latter represent their best interests. In no way can be thought that the only purpose of the law is to protect the interests of the physicians.

In 1981, it was enacted the Law 23, which is the current standard of medical ethics. Since then - 34 years later - customs in the scope of practice have substantially changed. Since customs are morally qualified and valued by society, moral must adjust its approach as those change. So it is then understandable that many of the moral standards referred to in the primal law have become obsolete. Whereas the test of time is the most severe and just laws judge, after nearly seven decades of Act 23 being tested, there have been revealed both its goodness and defects.

With the advent of bioethics in the 70s decade of last century, both the face and the brain of traditional ethics suffered fundamental changes. Taken as a specialty of bioethics, medical ethics also changed, to the point that its distance from the Hippocratic ethics - in effect for over twenty centuries - forced to reassess some of the principles that underpinned it.

For example, medical paternalism, the base of the Hippocratic Oath, was replaced by the patients' rights, invoking the moral and legal autonomy of them, forcing the physician to consider as *prima facie* duties, in addition to the autonomy duty, the beneficence and non-maleficence ones. Since the medical practice, which was private, became socialized; the doctor-patient relationship turned to be dependent on state and private intermediaries. Such interference was one of the customs introduced by the Law 100 of 1993. On the other hand, the society and the state, which were absent actors in the Hippocratic precepts, impelled by the principle of justice, rose health to the rank of fundamental right, so the State assume the duty to guarantee it, for which they made from the doctor an indispensable instrument to achieve it.

It is well known that the more they approach to perfection, the fairer the laws will be, more heeded, and more enduring. That is why doctors consider necessary that the Law 23 of 1981 suffered modifications aimed at rejuvenate it, i.e., to improve it, knowing that to achieve perfection is a factual impossible. Repeatedly, attempts were made to achieve that purpose, but the expected success could not be achieved.

Taking into account the successful experience gained by the so-called Great National Medical Board when proposing a reform of the health system through a statutory law, it was believed appropriate to follow the same strategy to reform the professional ethics code. This time, the Big Board was composed by one representative of each the National Academy of Medicine, the Colombian Medical Association, the Association of Scientific Societies, the Medical Federation, the Colombian Association of Faculties of Medicine, the National Medical Ethics Tribunal and the Colombian Institute of Bioethics Studies. It is easy to see that the work of this Board had the backing of the national medical staff. Aware of its responsibility to colleagues and to society as a whole, the Board dealt with the revision of the law in force, and then of a careful drafting a new text, so that the introduced changes would allow to have updated standards, in line with the time, providing the physicians with a correct medical practice. In other words, contributing to the proper performance of sensitive services that the society needs and expects from them.

The reform proposal - currently being considered by the Congress of the Republic - is original for the importance attached to moral principles set out clearly and with a pragmatic approach, knowing that these principles are the compass which will allow following the road map in a correct way. Besides, for the ones in charge of prosecuting their colleagues for alleged code violations, they are the obligatory point of reference to practice their magisterium with equity.

It has been a secular tradition that the neophytes in professional medical issues pledge before a competent authority their professional act, with the purpose of committing to exercise their profession properly. The Hippocratic Oath was considered for many centuries as the universal paradigm, to the extent that any technical or ethical deviation was regarded as a denial. Faced with changes in customs mentioned earlier, the Oath, which had religious connotations, has been replaced by a promise to society, which is the one to be served, and the one that, ultimately, dictates the rules of behavior (objective moral). In the reform proposal, the promise has been adjusted to the new customs.

After the above considerations, it is convenient to register a final thought, regarding the spirit that should accompany those who perform as doctors, a thought that subscribed the preamble that we delivered to the law makers. By meeting, the physicians, the rules written into this moral code, what they are serving is the coercive duty, the one that is imposed by society. If their consciences are not convinced of the goodness of these rules, they are not fulfilling their ethical duty, as the correction of acts that originate in the privacy of their consciousness, oblivious to external rewards or penalties. It is about the "subjective moral", which is what stamps the seal of ethics in our actions.

Finally, I wish to emphasize that the proposed reform of the Law on Medical Ethics is the product of the will and consensus of the national medical body, which, using the autonomy enshrined in the Statutory Health Law, gives authentic samples to the Colombian society that self-regulating their practice, the only intention is to become a pledge of warranty for it. 

